# Coping strategies and their association with diabetes specific distress, depression and diabetes self-care among people living with diabetes in Zambia

**DOI:** 10.1186/s12902-022-01131-2

**Published:** 2022-08-28

**Authors:** Given Hapunda

**Affiliations:** Department of Psychology, School of Humanities and Social Sciences, University of Zambia, P.O Box 32379, Lusaka, Zambia

**Keywords:** Coping strategies, Diabetes specific- distress, Depressive symptoms, Diabetes self-care, Type 1 and Type 2 diabetes

## Abstract

**Objectives:**

Utilising coping strategies to reduce and manage the intensity of negative and distressing emotions caused by diabetes is essential. However, little is known about the use of coping strategies among people living with diabetes in Sub-Saharan African countries like Zambia. This study investigates coping strategies used by people with diabetes in Zambia and how these are associated with diabetes-specific emotional distress, depression and diabetes self-care.

**Methods:**

Cross-sectional data from 157 people with diabetes aged between 12 and 68 years were collected. Of the 157, 59% were people with type 1 diabetes and 37% with type 2 diabetes. About 4% had missing information in their record but had either type 1 or type 2 diabetes. Coping styles were measured using the Brief Version of the Coping Orientation to Problems Experienced (Brief COPE), diabetes specific-distress using the Problem Areas in Diabetes, depression using the Major Depression Inventory and self-care using the Diabetes Self-Care scale.

**Results:**

Data showed that adaptive coping strategies such as religious coping, acceptance among others, were the most frequently used coping strategies among Zambian individuals with diabetes. Maladaptive coping strategies e.g., self-blame and self-distraction were related to increased diabetes specific-distress and depression. Emotional support was related to better diabetes self-care, while self-blame was related to poor diabetes self-care.

**Conclusion:**

There is need to help individuals with diabetes identify adaptive strategies that work best for them in order to improve their quality of life.

## Background

Diabetes self-care can be very complex, challenging and stressful [1, 2], especially in developing countries where health care and formal support systems are still underdeveloped [3]. Daily self-management involves frequent blood glucose monitoring and insulin adjustments and administration, engaging in physical activities, consideration of nutrition, managing sleep duration and blood glucose level. In addition to the medical, social, financial demands of optimal daily self-management, emotional problems are common and contribute to the overall burden of living with diabetes [4, 5]. Therefore, the goal for the treatment of diabetes is to prevent acute and chronic complications while preserving a better quality of life and psychological well-being [6]. The burden that comes with diabetes management is often stressing. Therefore, it is not surprising that diabetes management is associated with diabetes specific-distress. [7–10] Diabetes specific-distress is defined as emotional distress associated with the ongoing worries, burdens and concerns that occur when managing a demanding chronic disease like diabetes over time [11].

Diabetes is a complex disease to manage. Comorbid complications and conditions such as hypoglycaemia, hyperglycaemia, neuropathy, nephropathy, and retinopathy are also common in people with diabetes. Hence, there is need for people with diabetes to find optimal ways to manage the condition. [4, 12]. Such conditions negatively affect the quality of life of diabetes people. Already, evidence in Zambia shows that healthy controls (22.42 vs 18.58) had better scores on life satisfaction than young people with type 1 diabetes [13]. To help people with diabetes cope, adaptive coping strategies are important. An adaptive coping strategy is a technique that an individual uses to help him or her to adjust adequately or appropriately to the situation or stressor that requires one to manage, and the opposite of this is what is known as maladaptive [14]. Adaptive coping strategies such as acceptance and active coping can help maintain good health outcomes, such as glycaemic control [15]. In contrast, maladaptive strategies such as wishful thinking and avoidant can affect metabolic control and psychosocial outcomes such as quality of life and depressive symptoms [15–17]. Adaptive and maladaptive coping strategies can either be problem-focused or emotional-focused. Problem-focused coping refers to efforts directed toward rational management of a problem, and it is aimed at changing the situation causing distress whereas emotion-focused coping pertains to efforts to reduce emotional distress caused by the stressful event and manage or regulate emotions that might accompany or result from the stressor. Behavioural coping strategies are used by a person to manage a stressful event or situation by modifying his or her actions [18].

Diabetes specific psychosocial challenges are prevalent in people with type 1 and type 2 diabetes [16, 19]. For instance, the 2018 clinical consensus guidelines on paediatric diabetes highlight that children and adolescents with type 1 diabetes experience depression, diabetes-specific distress, stress, cognitive and school performance challenges, eating disorders, low general and diabetes-specific quality of life compared to their healthy counterparts [20]. Evidence from a systematic review also indicated that anxiety, depression, stress and diabetes-specific distress are the key influential psychosocial factors that determine the psychological wellbeing of people living with type 2 diabetes [16]. Further, anxiety and disease-related distress are known to be problematic for people with type 1 and type 2 diabetes [16]. In Zambia, levels of diabetes-specific distress (33.8 ± 27.2) and severe depressive symptoms (10.8%) have been reported among type 1 and type 2 diabetes in Zambia [21]. On its own, diabetes can be an unpredictable and stressful disease [16].

Data from a 14-country study with about 10 countries sampled from low- and middle-income countries (LMICs), of which two (Uganda and Kenya) were from SSA, showed that in many of these LMICs, diabetes care was not comprehensive. Most services lacked appropriate identification and care processes for psychological and psychiatric problems [22]. This is because, in developing countries, the medical care value chain does not include mental health specialists due to a shortage of such expertise. This is despite the evidence that psychosocial issues contribute to the development of diabetes, especially type 2 and that diabetes itself contributes to the development of psychosocial problems in individuals with diabetes. Thus, if service care providers simultaneously care for psychological well-being and medical outcomes, their patients’ outcomes will also get better [23]. Despite this evidence in Sub Saharan African, little is known on the use of coping strategies in individuals with diabetes. Thus, coping strategies remain an important study area, especially in developing countries with unique social-cultural factors, suboptimal diabetes care and management systems.

A stressor’s effect on a person is based on that person’s feeling of threat, vulnerability, and ability to cope than the stressful event itself [24]. How one manages the stressor can be adaptive or maladaptive. Coping is maladaptive when an individual applies techniques that are not adequate or appropriate to the situation or stressor that requires one to manage [14]. Greater engagement in maladaptive coping is associated with anxiety, depressive symptoms and poorer quality of life [25, 26]. Evidence from qualitative data among Zambian adolescents with type 1 diabetes showed young people used maladaptive coping strategies such as avoidance. Specifically, evidence showed that young people avoiding injecting themselves with insulin was common [27]. The problem-focused and the emotional- focused coping strategies are the two common types of personal coping strategies. The problem-focused coping strategies are aimed at changing the source of stress, while emotional focused are oriented towards managing the emotions that accompany the perceived stressor. Both are effective in making the stressed individual feel better but are not equally effective in managing stress. In diabetes, individuals with emotion-focused, social-support focused and problem-focused coping have higher levels of positive diabetes self-care activities while individuals with avoidance-focused coping had negative diabetes self-care activities [15]. Although some emotional and behavioural strategies are considered maladaptive, in situations where the stressor is unavoidable, such as diabetes self-care, maladaptive strategies, whether emotional or behavioural, can be an option [28]. For instance, distraction and religion as coping strategies do not remove the stressor but contribute to higher wellbeing and positive emotions [28]. Still, it can be helpful in diabetes as it empowers an individual and can lead to finding meaning and purpose in disorder. In light of the Brief Coping Orientation to Problems Experienced (Brief-COPE), the primary tool of this study, planning, active coping, positive reframing, acceptance, are among those considered adaptive coping strategies. Self-distraction, denial, behavioural disengagement, venting, humour, self-blame and substance use are examples of maladaptive coping strategies [29].

## Methods

### Study aim

This study aimed to identify which coping strategies are used by Zambian with type 1 and type 2 diabetes and examine how these strategies are associated with diabetes-specific distress, depression, and diabetes self-care activities.

### Design

A cross-sectional study was conducted among outpatients with type 1 or 2 diabetes mellitus. Participants were from four major hospitals in Zambia, with different age groups and socioeconomic backgrounds.

### Participants and sites

The study participants were individuals with type 1 and type 2 diabetes recruited from four major hospitals in Zambia, namely Lusaka, Kitwe, Ndola and Livingstone. The sample included both adolescents according to WHO definition (Adolescence is the phase of life between childhood and adulthood, from ages 10 to 19) and adults. Purposive sampling was used to recruit respondents as long as they met the inclusion criteria of being at least 12 years or older, having been diagnosed with diabetes for at least six months, and using oral medication or insulin therapy. The questionnaires were administered during the routine appointments individuals with type 1 and 2 diabetes had with their respective health care providers. Data were collected using researcher assisted questionnaires from these four city hospitals over one year.

### Instruments

*Generic coping strategies (not diabetes-specific) *were assessed using the brief COPE. The brief COPE is a short version of the COPE developed by Carver, Scheiver & Weinbraub [29]. The 28 items self-report scale assesses coping styles or strategies on two main dimensions – problems focused and emotional focused coping styles. The scale consists of 14 domains/subscales (self-distraction, active coping, denial, substance use, use of emotional support, use of instrumental support, behavioural disengagement, venting, positive reframing, planning, humour, acceptance, religion, self-blame) of two items each. Participants respond to statements on a four Likert scale (1 = I have not been doing this, to 4 = I have been doing this). Higher scores indicate greater use of a particular coping strategy. The full scale has a good Cronbach’ alpha of 0.84 [29]. In the current study, Cronbach alphas were 0.81, and Lambda 2 was 0.82. The two-item based 14 sub-scales have previously been documented to have low reliabilities [30, 31]. In the current study, internal consistency was low, ranging for most scales 0.34–71. Alpha for subscales was as follows: self-distraction = 0.42; active coping = . 56; denial = 0.52; substance use = 0.71; use of emotional support 0.46; use of instrumental support = 0.55; behavioural disengagement = 0.34; venting = 0.40; positive reframing = 0.50; planning = 0.54; humour = 54; acceptance = 0.48; religion = 0.59; self-blame = 0.34. Nevertheless, the overarching copying style i.e., Problem focused, emotional focused and avoidant reliabilities are acceptable [32] and it is widely used for clinical practice.

*Diabetes-specific-distress* was assessed using the Problem Areas in Diabetes (PAID). The PAID is a 20-item self-report measure used to assess diabetes-specific distress, including a range of feelings such as diabetes-related anger, fear, depression, worry and guilt. Items can be responded to on a scale from 0 (not a problem) to 4 (serious problem). An overall score for the PAID can be calculated by adding all of the item scores and multiplying them by 1.25, which gives a total score ranging from 0–100. Higher scores indicate more diabetes specific-distress. Reported Cronbach’s alphas for the PAID ranges from 0.84 to 0.96 [33, 34]. In the current study the alpha was 0.88 (Lambda2 = 0.89).

*Diabetes Self-care* was assessed using the Self-Care Inventory. The 13 item Self-Care Inventory (SCI) is a self-report measure used to assess people’ perceptions of their adherence to diabetes self-care recommendations over the past month. Individuals rate themselves on a 5-point Likert scale that reflects how well they followed recommendations for self-care during the past month (i.e., 1 = “never do it” to 5 = “always do this as recommended, without fail”). Higher scores indicate more optimal diabetes self-care [35]. Cronbach’s alpha for the SCI was 0.84 (Lambda2 = 0.85) for type 1 diabetes and 0.85 (lambda2 = 0.86) for type 2 diabetes.

*Depression* was assessed using the Major Depression Inventory (MDI). The MDI is a 12-item self-report questionnaire used to assess depression. Items of the MDI ask individuals to rate how long in the past two weeks each of the depressive symptoms was present on a six-point scale ranging from 0 “not at all” to 5 ”all time. Therefore, a higher score indicates the presence of depressive symptoms. It can be used as an instrument measuring the severity of depression with a range from 0—60. In previous studies, the MDI had excellent internal consistency with Cronbach alphas ranging from 0.89 to 0.94 [36, 37]. In the current study Cronbach alpha was 0.80 (Lambda2 = 0.81).

### Data analysis

Descriptive statistics involving means (standard deviation) and frequency percentages were computed to identify the coping strategies frequently used by people in this study. Coping strategies used 50% or more were considered frequently (commonly) used. An independent *t*-test was computed to test whether the use of coping strategies differed between males versus females, type 1 versus type 2 diabetes, adolescents versus adults. Further, Pearson Product correlation was used to examine the association between coping strategies, diabetes-specific—distress, depression and diabetes self-care. All analysis was done in IBM SPSS version 23. Statistical significance was set at *p* < 0.05.

## Results

Data were collected from a total of 157 individuals with diabetes from four main hospitals in Zambia, namely Lusaka 48 (31%), Kitwe 60 (38%), Ndola 35 (22%) and Livingstone 14 (9%). One hundred and fifty-seven (157) respondents, of which 93 (59%) had type 1 diabetes mellitus, 80 (51%) females, and 42 (27%) were adolescents. The average age of the respondents was 39 ± 17 years. See Table 1 for the demographic characteristics of the study sample.

**Table 1:** Demographic and clinical characteristics of 157 participants with type 1 and type 2 diabetes

Sex, *n* (%)	
Females	80 (51%)
Age, mean (SD)	39 ± 17
Age range	12–68 years
Location of patients	
Lusaka	48 (31%)
Kitwe	60 (38%)
Ndola	35 (22%)
Livingstone	14 (9%)
Age Category *n* (%)	
Adolescents	42 (27%)
Adults	115 (73%)
Educational levels *n* (%)	
Adolescents (42)	
5-7^th^ Grade (Primary school)	14 (31%)
8-12^th^ Grade (Secondary school)	16 (38%)
Missing	14 (31%)
Adults (115)	
Primary education	10 (9%)
Secondary education	29 (25%)
Tertiary education	22 (19%)
Missing	54 (47%)
Marital status (Adults/115) *n* (%)	
Single	6 (5%)
Married	80 (70%)
Missing	29 (25%)
Type of diabetes	
Type 1	93(59)
Type 2	58 (37)
Missing (either type 1or 2)	6 (4)
BMI mean (*SD*)	25 (5) kg/m^2^
Males	25 (5) kg/m^2^
Females	26 (5) kg/m^2^
Adolescents	22 (4) kg/m^2^
Adults	27 (5) kg/m2

The most frequently used coping strategies among Zambian individuals with diabetes are presented in Table 2. In general, adaptive coping strategies (67% religious coping; 58% acceptance, 57% seeking instrumental support and 56% using active coping) were commonly used among Zambian individuals with diabetes. For details on strategies applied by males and females with type 1 and type 2, see Table 3.

**Table 2:** Proportion of individuals with diabetes using each of the fourteen coping strategies

**Coping strategy**		**Type of Diabetes**		**Age Category**		**Sex**	
	**All types**	**Type 1**	**Type 2**	**Adolescents**	**Adults**	**Males**	**Females**
**Adaptive coping strategies**							
Active coping	**84(56%)**	**47(53%)**	**34(62%)**	**15(38%)**	**69(63%)**	**43(61%)**	**40(52%)**
Emotional use	70(46%)	36(40%)	**31(53%)**	16(41%)	54(48%)	**40(53%)**	30(39%)
Instrumental use	**87(57%)**	**47(52%)**	**36(63%)**	**20(50%)**	**67(60%)**	**40(53%)**	**47(62%)**
Positive reframing	52(34%)	28(32%)	21(36%)	7(18%)	45(40%)	29(38%)	13(31%)
Planning	69(47%)	36(42%)	**30(53%)**	10(29%)	**59(52%)**	**38(69%)**	30(40%)
Acceptance	**85(58%)**	**50(59%)**	**41(56%)**	**23(64%)**	**62(56%)**	**43(60%)**	**41(56%)**
Religion	**101(66%)**	**56(62%)**	**41(71%)**	**21(53%)**	**80(70%)**	**49(64%)**	**51(66%)**
**Maladaptive coping strategies**							
Self-blame	12(8%)	9 (10%)	6(10%)	6(11%)	6(5%)	3(4%)	8(10%)
Self-distraction	49(33%)	28(32%)	21(37%)	12(31%)	37(34%)	23(31%)	26(34%)
Denial	27(18%)	13(14%)	13(22%)	5(12%)	22(19%)	12(16%)	5(19%)
Substance use	5(3%)	3(3%)	2(3%)	2(5%)	3((3%)	4(5%)	1(1%)
Behavioural disengagement	27(18%)	16(18%)	11(20%)	5(13%)	22(20%)	17(24%)	10(13%)
Venting	27(19%)	19(22%)	8(15%)	11(28%)	16(15%)	9(13%)	18(25%)
Humour	14(10%)	8(9%)	6(11%)	6(15%)	8(8%)	3(4%)	11(15%)

Proportion ≥ 50% were considered common coping strategies among diabetes individuals

**Table 3:** Mean differences of participant characteristic on different coping strategies

Coping strategy		Type of Diabetes		*P *Value	Age Category		*P *Value	Sex		*P *Value
		**T1D**	**T2D**		**Adolescents**	**Adults**		**Males**	**Females**	
Brief cope	M	53.40	57.18	0.21	50.65	55.95	0.14	56.15	53.40	0.35
	SD	15.96	13.77		16.65	14.39		17.09	12.47	
Behavioural strategies	M	27.10	29.20	0.25	24.21	29.13	**0.01**	29.79	26.30	0.42
	SD	9.47	9.52		9.61	9.08		10.48	7.88	
Emotional strategies	M	25.60	28.09	**0.04**	25.29	26.67	0.09	26.25	26.48	0.23
	SD	8.03	6.30		8.76	7.11		8.06	6.94	
**Adaptive coping strategies**										
Active coping	M	5.31	5.44	0.75	4.85	5.55	0.07	5.63	5.12	0.14
	SD	2.20	1.99		2.28	2.03		2.07	2.15	
Emotional support	M	4.48	4.95	0.21	4.41	4.76	0.40	4.91	4.49	0.25
	SD	2.13	2.27		2.31	2.21		2.11	2.32	
Instrumental support	M	5.24	5.74	0.15	5.20	5.54	0.36	5.40	5.51	0.73
	SD	2.13	2.27		2.31	2.21		2.11	2.32	
Positive reframing	M	3.98	4.17	0.61	3.45	4.33	**0.04**	4.28	3.96	0.40
	SD	2.29	2.26		2.19	2.30		2.42	2.18	
Planning	M	4.61	4.91	0.45	4.15	4.96	0.08	5.02	4.49	0.17
	SD	2.31	2.35		2.55	2.26		2.35	2.34	
Acceptance	M	5.76	5.61	0.67	5.72	5.70	0.96	5.75	5.63	0.72
	SD	2.03	2.11		2.21	2.03		1.97	2.17	
Religion	M	5.60	6.03	0.22	**5.18**	**6.00**	**0.03**	5.46	6.09	0.06
	SD	2.13	1.98		**2.50**	**1.90**		2.20	1.93	
**Maladaptive coping strategies**										
Denial	M	2.76	3.29	0.19	2.54	3.05	0.25	2.85	3.01	0.69
	SD	2.40	2.45		2.46	2.45		2.51	2.40	
Substance use	M	0.91	0.81	0.75	0.73	0.92	0.56	1.10	0.66	0.13
	SD	1.90	1.72		1.96	1.77		2.07	1.53	
Behavioural disengage	M	2.95	3.09	0.74	3.67	3.04	0.41	3.11	2.79	0.42
	SD	2.39	2.38		2.31	2.41		2.68	2.09	
Venting	M	3.26	3.53	0.51	3.40	3.23	0.69	3.18	3.37	0.63
	SD	2.45	2.08		2.63	2.21		2.29	2.39	
Humour	M	1.90	1.62	0.45	1.95	1.67	0.48	1.55	1.97	0.23
	SD	2.15	2.09		2.56	1.91		1.86	2.33	
Self-blame	M	2.44	1.90	0.13	**2.73**	**1.96**	**0.04**	2.16	2.08	0.81
	SD	2.34	1.81		**2.53**	**1.97**		1.91	2.28	

Although there were differences in the use of coping strategies between type 1 and type 2 diabetes individuals, adolescents and adults, and females and males, most of these differences were not statistically significant. The only coping strategy that was significant was the use of religion between adults [70% (6.00 ± 1.90)] and adolescents [53% (5.18 ± 2.50)], *p* < 0.05. The other coping strategy that was statistically significant was the use of self-blame with [11% (2.75 ± 2.53)] of adolescents vs [5% (1.96 ± 1.97)], *p* < 0.05 of adults reporting using it. Use of positive reframing was also significantly different between adolescents [18% (3.45 ± 2.19)] vs [40% (4.33 ± 2.30)], *p* < 0.05. Overall, a significant difference in the use of emotional strategies emerged between individuals with type 1 diabetes (25.60 ± 8.03) and individuals with type 2 diabetes (28.09 ± 6.30), *p* < 0.05. Further, there was a significant difference in the use of behavioural strategies between adolescents (24.21 ± 9.61) vs adults with diabetes (29.13 ± 9.08), *p* < 0.01. For details see Table 3.

Overall, Zambian individuals with diabetes reported using more behavioural focused strategies 20.08 ± 9.28 than emotional strategies 26.60 ± 7.46 (*t* = 2.25, df = 106, *p* < 0.05). Among adult people, behavioural strategies were more used 29.13 ± 9.08 compared to emotional strategies 26.67 ± 7.11 (*t* = 2.88, df 82, < 0.01) while for adolescents, emotional strategies were more likely to be used (25.29 ± 8.76) compared to behavioural strategies (24.21 ± 9.61). However, these differences were not statistically significant, *t* = -0.78, df, 23, *p* > 0.05. Regarding the type of diabetes, behavioural strategies were likely to be used 27.10 ± 9.47 compared to emotional 25.60 ± 8.03 among individuals with type 1 diabetes, although this difference was not statistically significant *t* = 1.66, df = 57, *p* > 0.05. For type 2 diabetes, behaviour strategies were more likely to be used 29.20 ± 9.52 compared to emotional strategies 28.09 ± 6.30, although this difference was also not statistically significant. For males, behaviour strategies were more likely to be used 29.79 ± 10.48 compared to emotional strategies 26.25 ± 8.06 and this difference was statistically significant *t* = 2.16, df = 52, *p* < 0.01. For females, there was no difference in the use of behavioural strategies (26.30 ± 7.88) and emotional strategies 26.48 ± 6.94), *t* = -0.20, df = 53, *p* > 0.05.

Correlations between coping strategies, diabetes specific-distress, depression and diabetes self-care are presented in Table 4. Results showed that use of behavioural focused strategies was associated with increased report of diabetes specific-distress (*r* = 0.430, *p* < 0.001), and high use of emotional focused strategies was also associated with increase report of specific-distress (*r* = 0.374, *P* < 0.001) and with increased report of depressive symptoms (*r* = 0.475, *p* < 0.001).

**Table 4:** Correlation matrix for coping strategies, diabetes specific-distress, depression and diabetes self-care

	**Diabetes Specific Distress**	**Depression**	**Self-Care**
Brief Cope (Total Score)	0.475***	0.246*	0.007
Behavioural focused	0.430***	0.175	0.062
Emotional focused	0.374***	0.321**	0.003
***Adaptive coping strategies***			
Active coping	0.247**	0.108	0.078
Use of emotional support	0.079	0.141	0.263**
Use of instrumental support	0.159	-0.039	0.118
Positive reframing	0.278**	0.142	0.104
Planning	0.120	-0.001	-0.017
Acceptance	-0.053	0.003	-0.016
Religion	0.122	0.102	0.129
***Maladaptive coping strategies***			
Self-blame	0.238**	0.150	-0.171*
Self- distraction	0.370***	0.137	0.017
Denial	0.402***	0.191*	-0.071
Substance use	0.193*	0.177*	-0.066
Behavioural disengagement	0.448***	0.170	-0.023
Venting	0.326***	0.206*	0.068
Humour	0.173*	0.230**	-0.016
	*p = < 0.05	**p = < 0.01	***p = < 0.001

Adaptive coping strategies including active coping (*r* = 0.247, *p* < 0.01) and positive reframing (*r* = 0.278, *p* < 0.01) were associated with increased specific-distress while use of emotional support was only associated with improved diabetes self-care (*r* = 0.263, *p* < 0.01).

Maladaptive coping strategies including self-distraction (*r* = 0.370, *p* < 0.001); behavioural disengagement (*r* = 0.448, *p* < 0.001); denial (*r* = 0.402, *p* < 0.001); substance use (*r* = 0.193, *p* < 0.05); humour (*r* = 0.173, *p* < 0.05); self-blame (*r* = 0.238, *p* < 0.01) and; venting (r = 0.326, *p* < 0.001) were associated with increased specific-distress. In addition, Denial (*r* = 0.191, *p* < 0.05); substance use (*r* = 0.177, *p* < 0.05); self-blame (*r* = 0.230, *p* < 0.01) and; venting (*r* = 0.206, *p* < 0.05) were associated with increased depressive symptoms. Furthermore, use of self-blame (*r* = -0.171, *p* < 0.05) was associated with poor diabetes self-care.

## Discussion

This study aimed to identify coping strategies mostly used among individuals with diabetes in Zambia and examine how these strategies are associated with diabetes specific-distress, depression, and diabetes self-care. This study showed that the most frequently used coping strategies were religion, active coping, instrumental use and acceptance. These strategies are considered adaptive in light of people with chronic diseases such as diabetes and non-clinical samples. These findings are similar to a Turkish study of type 1 and 2 diabetes except for active coping. The differences included positive reframing, self-distraction, and venting common in a Turkish [6] compared to the Zambian sample. These findings could be different from people in Western developed countries.

The highest proportion of individuals used religious coping. Zambia is a very religious country. Therefore, most Christians hope God to heal them or ameliorate their suffering by relying on inner strength to keep them going with hope, similar to findings in Iran [38]. Consistent with other chronic illnesses such as HIV, there are reports of individuals relying on inner strength supplied by their Christian faith to cope with HIV [39].

In addition to frequently using adaptive coping strategies, Zambian individuals with diabetes were more likely to use behavioural than emotional strategies. Behavioural coping strategies are overt physical activities aimed at removing or averting the stressor. In contrast, emotional strategies aim to reduce and manage the intensity of the negative and distressing emotions caused by a stressful situation rather than solving the problematic situation itself [40]. Thus, emotional strategies make a person feel better but don’t solve the source of one’s emotional distress. Emotional focused coping often gets utilised when the problem is out of control or in situations where the stressor is unavoidable. For example, having a chronic and terminal illness or sudden death, and one needs to cope and accept the situation [40]. Furthermore, adults were more likely to use behavioural strategies than adolescents to use behavioural strategies. It is under this strategy that problem solving skills like planning have been found to be a common skill taught in a variety of patient education and self-management interventions [41] Thus, teaching coping and problem-solving skills may improve quality of life and diabetes management [41].

Mixed results were found regarding the association between coping strategies and diabetes specific-distress, depression and self-care. Although individuals with diabetes used adaptive coping strategies more than maladaptive, most of them were not associated with psychological outcomes except for active coping and emotional support. Adaptive coping strategies confront problems directly, make practically realistic evaluations of problems, recognise and change unhealthy emotional reactions, and prevent adverse effects on the body. According to Carver, people who use adaptive strategies (active coping, use of informational support, planning, and positive reframing) use them to change the stressful situation [29]. For example, individuals with diabetes may apply adaptive strategies to change treatment and food related problems that they might find distressing. Surprisingly, active coping where one is intentionally or is goal-directed to minimise the physical, psychological or social stressor was associated with increased diabetes specific-distress. It could be that some of the stressors, such as deprivation of food or being overwhelmed with diabetes regimens, cannot be removed because they are beyond their capabilities. It can be exasperating to actively cope with what one cannot change (or reduce) given the high poverty and weak health system these people find themselves in. This could explain why many individuals use emotional focused coping, which gets utilised when the problem is out of one’s control. Unlike the adaptive (problem) focused strategies (venting, use of emotional support, humour, acceptance, self-blame, and religion), emotional strategies aim to regulate emotions associated with the stressful situation [29]. Distressing emotions such as worrying about low sugar levels, fear of future complications and feeling guilty when off-track with diabetes management are common in patients, and the use of emotional strategies to cope with such emotions is high, as found by this study. Further, in the face of insufficient manageability of a stressor, some individuals may decide to use maladaptive strategies instead because the conventional resources of help seem to be exhausted [42]. Thus, a person needs to cope and accept the situation beyond their capability.

Religion and spirituality are commonly employed as coping mechanisms in individuals with diabetes [43]. Although religion was the most frequently used coping strategy, it was not associated with diabetes specific-distress, depression and diabetes self-care. Our findings contrast with a sample of Nigerian individuals with diabetes whose high intrinsic and extrinsic religiosities were associated with positive coping skills and better treatment outcomes in people with depression or diabetes [44]. The Nigerian findings confirm that religion is an adaptive coping strategy for diabetes. In our study, we only found a significant difference in the use of religion as a coping strategy between adolescents and adults. This finding is consistent with the trends worldwide that shows that young people are less religious than adults [45]. This finding suggests that adults compared to adolescents use emotional strategies to regulate emotions associated with their diabetes stressful situations.

Regarding diabetes self-care, only emotional support was associated with better diabetes care. Emotional and psychological support has been documented to improve people’s ability to adjust or take adequate responsibility in diabetes self-management [46]. Evidence from an international observation study, consistent with the current study showed that increased emotional support was associated with better diabetes self-care [47]. In chronic illnesses such as diabetes, emotional support is considered an adaptive coping strategy. Specifically, it positively impacts a healthy diet, increased perceived support, higher self-efficacy, improved psychological well-being, and better glycaemic control, according to data from a recent systematic review [48]. Although emotional support as a coping strategy was not significantly different between adults and adolescents, adults used emotional support more than adolescents to cope with stressors. While both adolescents and adults need emotional support, emotional support must not be seen as controlling or intrusive in adolescents. Self-blame was associated with reduced diabetes self-care. Consistent with other studies, self-blame has been associated with negative health outcomes [49]. As observed earlier, self-blame is related to emotional distress, affecting diabetes self-care [21, 50]. This is because self-criticism or blame is associated with low resilience to adhere to diabetes care [51] hence considered a maladaptive coping strategy.

As expected, most maladaptive coping strategies were associated with negative outcomes. For instance, some scales within behavioural focus such as self-distraction, substance use, and behavioural disengagement were associated with increased diabetes specific distress because they all temporary address the stressor. These do not take the problem away. These are also known as avoidant strategies in which physical or cognitive effort to disengage from the stressor is applied [29]. For example, with behavioural disengagement, individuals reduce their efforts to deal with the stressor, which maintains or increases diabetes specific-distress. Further, emotionally focused strategies were associated with increased diabetes specific-distress. This domain is a facet that has several maladaptive strategies such as denial, venting or humour which could worsen diabetes specific-distress. Therefore, it was not surprising that self-blame, humour, venting, and denial were associated with increased diabetes specific-distress. For instance, a systematic review of chronic conditions including diabetes found that self-blame for the onset for the diseases was associated with increased emotional distress [52]. Generally, our data is similar to the Turkish data that showed that problem-focused and emotional-focused strategies were used in both type 1 and type 2 diabetes [6].

Furthermore, denial, substance use, venting, self-distraction, and humour increased depressive symptoms. About 18% (14% type 1 and 22% type 2) used denial. Refusing to accept a problem or reality can interfere with one’s ability to tackle challenges simply because one cannot acknowledge the problem and downplay consequences which can, in the long run, increase anxiety if the problem is not going away. Moreover, people with depression in stressful situations often use strategies based on denial and avoidance and have more difficulties finding positive aspects of stressful events [53]. With regard to substance use, our study is consistent with findings of meta-analysis studies that have shown that depression is associated with concurrent alcohol use, drug use and impairment in clinical and community samples [54, 55]. Although the percentage of users was small, more education is needed. Only 3% (3% type 1 and 3% type 2) used this strategy. Humour was associated with increased depressive symptoms. About 10% (9% type 1 and 11% type 2) used this strategy. One possible explanation for this association is that humour is likely to increase depression if it is targeted at mocking a stressor, simply because humour works well as a coping strategy if the event or interaction is pleasurable [56]. Our findings are consistent with Kuiper and colleagues who showed that the maladaptive components of humour that are self-focused (e.g., self-defeating and belaboured humour) predict detrimental effects on poorer self-esteem, greater depression and anxiety [57]. Evidence from health care professionals’ interviews also suggests that humour in a therapeutic situation should be used in moderation and under certain socio-cultural conditions if it is to be effective [58]. In a disease like diabetes, it might not work. As with self-distraction, it was expected that this relationship would be observed.

Distracting oneself from a stressor is a temporal solution because the stressor does not go away with this strategy. Therefore, by temporarily distracting yourself, you may give the emotion some time to decrease in intensity, but it will still emerge later, which can be depressing. We observed that a good percentage of our sample utilises this strategy (35% [32% type 1 and 37% type). This observation is consistent with our initial qualitative study, which showed adolescents used distraction as a coping strategy [27]. Thus, the current study validates the previous qualitative study.

Venting was also associated with increased depressive symptoms. About 19% (22% type 1 and 15% type 2) used this strategy. Venting refers to stating unpleasant feelings or expressing one’s negative feelings [29] and is a maladaptive strategy. This coping strategy is similar to the explosiveness of speech, one of the Types A behaviour pattern characteristics. The Type A behaviour pattern is another response to a stressor characterised by extreme hostility, competitiveness, hurry, impatience, restlessness, aggressiveness, and explosiveness of speech [59]. This kind of behaviour is likely to increase sadness and loneliness, precursors of depression.

All coping scales (total score) were related to increased diabetes specific-distress and increased depressive symptoms. This finding remains unclear why. However, it could be that using multiple coping strategies can sometimes be frustrating when a person has not mastered the ones that work well for them, hence the increase in experience of diabetes-related stress and depressive symptoms. Consequently, using multiple styles that a person does not find effective may lead to a pessimistic outlook on finding a solution [60]. One has to identify what works to be consistently used and produce results. Using multiple coping strategies can lead to trouble organising thoughts on what works and does not work and may lead individuals to keep switching strategies to find what work. This process may be frustrating and increase diabetes specific-distress.

Moreover, emotional oriented coping appears to play a role in developing depressive symptoms, anxiety and diabetes specific-distress [61]. Equally, behavioural-focused strategies were associated with increased experience of diabetes specific-distress. Again, we believe that individuals using behavioural focused strategies apply coping flexibility (i.e., an individual’s ability to modify and change coping strategies depending on the context). The availability of numerous coping strategies if one has mastered their effectiveness may be an important precursor to coping flexibility. Coping flexibility can only be exercised if an individual can access and use different coping strategies [62] but may be ineffective if they do not master the strategies that work for them.

This study has some limitations. Firstly, we did not have data on diabetes biological markers such as HbA1c and specific medication the people were using. Secondly, our sample size was small. However, this study compared strategies used between type 1 and type 2 people, which most studies tend to report separately, making it difficult to make comparisons. Further, to the author’s best knowledge, this is the first study to investigate coping strategies that people with diabetes use in Zambia. Thus, this data on different coping strategies used between different age groups, types of diabetes, sex, and how they are used on different psychological challenges they face may be important for diabetes care and education in Zambia and other Sub-Saharan African countries.

## Conclusion

In conclusion, adaptive coping strategies such as religion, acceptance, instrumental support and active coping are the most frequently used coping strategies among Zambian individuals with diabetes. The difference only lay in the use of religion, with more adults likely to use it as a coping strategy. Mixed results were found on the use of coping strategies and how they are associated with diabetes-specific distress, depression and diabetes self-care. Some people used maladaptive coping strategies that affected their psychological well-being and diabetes management. There is need to help people use more behavioural but adaptive strategies in order to improve their quality of life.

## Data Availability

The datasets used and/or analysed during the current study available from the corresponding author on reasonable request. Data is not currently public because more analysis is still being conducted on the data set.

## Abbreviations

Brief COPE: Brief Coping Orientation to Problems Experienced
HIV: Human Immunodeficiency Virus
LMIC: Low- and middle-income country
MDI: Major Depression Inventory
PAID: Problem Areas in Diabetes
SCI: Self Care Inventory
T1D: Type 1 Diabetes
T2D: Type 2 Diabetes

## References

[1] Chan J, DeMelo M, Gingras J, Gucciardi E (2015). Challenges of diabetes self-management in adults affected by food insecurity in a large urban centre of Ontario Canada. Int J Endocrinol.

[2] Debussche X, Balcou-Debussche M, Besançon S, Traore SA (2009). Challenges to diabetes self-management in developing countries. Diabetes Voice.

[3] Pastakia SD, Pekny CR, Manyara SM, Fischer L (2017). Diabetes in sub-Saharan Africa–from policy to practice to progress: targeting the existing gaps for future care for diabetes. Diabetes Metab Syndr Obes.

[4] Hilliard ME, De Wit M, Wasserman RM, Butler AM, Evans M, Weissberg-Benchell J, Anderson BJ (2018). Screening and support for emotional burdens of youth with type 1 diabetes: strategies for diabetes care providers. Pediatr Diabetes.

[5] Sumlin LL, Garcia TJ, Brown SA, Winter MA, Garcia AA, Brown A, Cuevas HE (2014). Depression and adherence to lifestyle changes in type 2 diabetes: a systematic review. Diabetes Educ.

[6] Tuncay T, Musabak I, Gok DE, Kutlu M (2008). The relationship between anxiety, coping strategies and characteristics of patients with diabetes. Health Qual Life Outcomes.

[7] Delahanty L, Grant RW, Wittenberg E, Bosch JL, Wexler DJ, Cagliero E, Meigs JB (2007). Association of diabetes-related emotional distress with diabetes treatment in primary care patients with type 2 diabetes. Diabet Med.

[8] Strandberg RB, Graue M, Wentzel-Larsen T, Peyrot M, Rokne B (2014). Relationships of diabetes-specific emotional distress, depression, anxiety, and overall well-being with HbA1c in adult persons with type 1 diabetes. J Psychosom Res.

[9] Bronner MB, Peeters MA, Sattoe JN, van Staa A (2020). The impact of type 1 diabetes on young adults’ health-related quality of life. Health Qual Life Outcomes.

[10] Holt RI, DeVries JH, Hess-Fischl A, Hirsch IB, Kirkman MS, Klupa T, Ludwig B, Nørgaard K, Pettus J, Renard E, Skyler JS (2021). The management of type 1 diabetes in adults. A consensus report by the American Diabetes Association (ADA) and the European Association for the Study of Diabetes (EASD). Diabetes Care.

[11] Fisher L, Gonzalez JS, Polonsky WH (2014). The confusing tale of depression and distress in patients with diabetes: a call for greater clarity and precision. Diabet Med.

[12] Hema DA, Roper SO, Nehring JW, Call A, Mandleco BL, Dyches TT (2009). Daily stressors and coping responses of children and adolescents with type 1 diabetes. Child Care Health Dev.

[13] Malipa MN, Menon JA (2013). The relationship between compliance and quality of life among adolescents with diabetes mellitus type1. Med J Zambia.

[14] Kirby R, Shakespeare-Finch J, Palk G (2011). Adaptive and maladaptive coping strategies predict posttrauma outcomes in ambulance personnel. Traumatology.

[15] Albai A, Sima A, Papava I, Roman D, Andor B, Gafencu M (2017). Association between coping mechanisms and adherence to diabetes-related self-care activities: a cross-sectional study. Patient Prefer Adherence.

[16] McCoy MA, Theeke LA (2019). A systematic review of the relationships among psychosocial factors and coping in adults with type 2 diabetes mellitus. Int J Nurs Sci.

[17] Grey M (2011). Coping skills training for youths with diabetes. Diabetes Spectrum.

[18] David R, Weber JN. Diabetes Mellitus in Children. In: Kliegman RM, Geme JWS, Blum NJ, Shah SS, Tasker RC, Wilson KM, editors. Nelson textbook of pediatrics. 21th ed. Philadelphia: Elsevier; 2020. p. 3019–52.

[19] Young-Hyman D, De Groot M, Hill-Briggs F, Gonzalez JS, Hood K, Peyrot M (2016). Psychosocial care for people with diabetes: a position statement of the American Diabetes Association. Diabetes Care.

[20] Delamater AM, de Wit M, McDarby V, Malik JA, Hilliard ME, Northam E, Acerini CL (2018). ISPAD Clinical Practice Consensus Guidelines 2018: psychological care of children and adolescents with type 1 diabetes. Pediatr Diabetes.

[21] Hapunda G, Abubakar A, Pouwer F, Van de Vijver F (2016). Validity and reliability of the Zambian version of the Problem Areas in Diabetes (PAID) scale: a triangulation with cognitive interviews. South Afr J Diabetes Vasc Dis.

[22] Lloyd CE, Nouwen A, Sartorius N, Ahmed HU, Alvarez A, Bahendeka S, Basangwa D, Bobrov AE, Boden S, Bulgari V, Burti L (2018). Prevalence and correlates of depressive disorders in people with Type 2 diabetes: results from the International Prevalence and Treatment of Diabetes and Depression (INTERPRET-DD) study, a collaborative study carried out in 14 countries. Diabet Med.

[23] Clay RA (2017). More is needed to treat diabetes. Monit Psychol.

[24] Brannon L, Feist J (2007). Introduction to health psychology. India Edition, Thompson Wadsworth.

[25] Knowles SR, Apputhurai P, O’Brien CL, Ski CF, Thompson DR, Castle DJ (2020). Exploring the relationships between illness perceptions, self-efficacy, coping strategies, psychological distress and quality of life in a cohort of adults with diabetes mellitus. Psychol Health Med.

[26] Yasui-Furukori N, Murakami H, Otaka H, Nakayama H, Murabayashi M, Mizushiri S, Matsumura K, Tanabe J, Matsuhashi Y, Yanagimachi M, Nakamura K (2019). Coping behaviors and depressive status in individuals with type 2 diabetes mellitus. Ann Gen Psychiatry.

[27] Hapunda G, Abubakar A, Van de Vijver F, Pouwer F (2015). Living with type 1 diabetes is challenging for Zambian adolescents: qualitative data on stress, coping with stress and quality of care and life. BMC Endocr Disord.

[28] Waugh CE, Shing EZ, Furr RM (2020). Not all disengagement coping strategies are created equal: positive distraction, but not avoidance, can be an adaptive coping strategy for chronic life stressors. Anxiety Stress Coping.

[29] Carver CS (1997). You want to measure coping but your protocol’too long: Consider the brief cope. Int J Behav Med.

[30] Snell DL, Siegert RJ, Hay-Smith EJ, Surgenor LJ (2011). Factor structure of the Brief COPE in people with mild traumatic brain injury. J Head Trauma Rehabil.

[31] Yusoff N, Low WY, Yip CH (2010). Reliability and validity of the Brief COPE Scale (English version) among women with breast cancer undergoing treatment of adjuvant chemotherapy: a Malaysian study. Med J Malaysia.

[32] Poulus D, Coulter TJ, Trotter MG, Polman R (2020). Stress and coping in esports and the influence of mental toughness. Front Psychol.

[33] Welch GW, Jacobson AM, Polonsky WH (1997). The problem areas in diabetes scale: an evaluation of its clinical utility. Diabetes Care.

[34] Weissberg-Benchell J, Antisdel-Lomaglio J (2011). Diabetes-specific emotional distress among adolescents: feasibility, reliability, and validity of the problem areas in diabetes-teen version. Pediatric Diabetes.

[35] Weinger K, Butler HA, Welch GW, La Greca AM (2005). Measuring diabetes self-care: a psychometric analysis of the self-care inventory-revised with adults. Diabetes Care.

[36] Bech P, Rasmussen NA, Olsen LR, Noerholm V, Abildgaard W (2001). The sensitivity and specificity of the major depression inventory, using the present state examination as the index of diagnostic validity. J Affect Disord.

[37] Cuijper P, Dekker J, Noteboom A, Smit N, Peen J. The sensitivity and specificity of the Major Depression Inventory in outpatients. BMC Psychiatry, 2007; 7(39). 10.1186/1471-244X-7-39. 10.1186/1471-244X-7-39 PMC1973070 17688685

[38] Shamsalinia A, Pourghaznein T, Parsa M (2016). The relationship between hope and religious coping among patients with type 2 diabetes. Global J Health Sci.

[39] Chinouya M, O’Keefe E (2005). God will look after us: Africans, HIV and religion in Milton Keynes. Diversi Health Soc Care.

[40] Galor S. Emotional coping strategies. Amsterdam; 2012. Available from: https://drsharongalor.wordpress.com/2012/03/31/emotion-focused-coping-strategies/. [Updated 2012; cited 2021 November 7].

[41] Fisher EB, Thorpe CT, McEvoy DeVellis B, DeVellis RF (2007). Healthy coping, negative emotions, and diabetes management. Diabetes Educ.

[42] Büssing A, Ostermann T, Neugebauer EA, Heusser P (2010). Adaptive coping strategies in patients with chronic pain conditions and their interpretation of disease. BMC Public Health.

[43] Sridhar GR (2013). Diabetes, religion and spirituality. Int J Diabetes Dev Ctries.

[44] Amadi KU, Uwakwe R, Odinka PC, Ndukuba AC, Muomah CR, Ohaeri JU (2016). Religion, coping and outcome in out-patients with depression or diabetes mellitus. Acta Psychiatr Scand.

[45] Pew Research Centre. The age gap in religion around the world. Washington DC; 2018. Available from https://www.pewforum.org/2018/06/13/the-age-gap-in-religion-around-the-world/. [Updated 2018; cited 2021 November 7].

[46] Kalra S, Jena BN, Yeravdekar R (2018). Emotional and psychological needs of people with diabetes. Indian J Endocrinol Metab.

[47] Koetsenruijter J, van Eikelenboom N, van Lieshout J, Vassilev I, Lionis C, Todorova E, Portillo MC, Foss C, Gil MS, Roukova P, Angelaki A (2016). Social support and self-management capabilities in diabetes patients: an international observational study. Patient Educ Couns.

[48] Pamungkas RA, Chamroonsawasdi K, Vatanasomboon P (2017). A systematic review: family support integrated with diabetes self-management among uncontrolled type II diabetes mellitus patients. Behav Sci.

[49] Harry KM. The Cardiac Self-Blame Attributions scale as a predictor of physical and mental health outcomes in underrepresented patients with cardiovascular disease. University of Missouri-Kansas City; 2018.

[50] Khan A, Choudhary P (2018). Investigating the association between diabetes distress and self-management behaviors. J Diabetes Sci Technol.

[51] Yi-Frazier JP, Yaptangco M, Semana S, Buscaino E, Thompson V, Cochrane K, Tabile M, Alving E, Rosenberg AR (2015). The association of personal resilience with stress, coping, and diabetes outcomes in adolescents with type 1 diabetes: Variable-and person-focused approaches. J Health Psychol.

[52] Callebaut L, Molyneux P, Alexander T (2017). The relationship between self-blame for the onset of a chronic physical health condition and emotional distress: A systematic literature review. Clin Psychol Psychother.

[53] Orzechowska A, Zajączkowska M, Talarowska M, Gałecki P (2013). Depression and ways of coping with stress: a preliminary study. Med Science Monit.

[54] Conner KR, Pinquart M, Gamble SA (2009). Meta-analysis of depression and substance use among individuals with alcohol use disorders. J Subst Abuse Treat.

[55] Conner KR, Pinquart M, Duberstein PR (2008). Meta-analysis of depression and substance use and impairment among intravenous drug users (IDUs). Addiction.

[56] Nezlek JB, Derks P (2001). Use of humor as a coping mechanism, psychological adjustment, and social interaction. Humor.

[57] Kuiper NA, Grimshaw M, Leite C, Kirsh G. Humor is not always the best medicine: Specific components of sense of humor and psychological well-being. 2004;17(12):135–168. 10.1515/humr.2004.002.

[58] de Almeida CV, Nunes C (2020). Humour is important in healthcare relationship?. Open Access Libr J.

[59] Ito M, Matsushima E (2017). Presentation of coping strategies associated with physical and mental health during health check-ups. Community Ment Health J.

[60] Sahler OJ, Carr JE. Coping strategies. In Carrey WB, editor. Developmental behavioral pediatrics. Philadelphia: W.B. Saunders; 2009. p. 491–6.

[61] Burns RJ, Deschênes SS, Schmitz N (2016). Associations between coping strategies and mental health in individuals with type 2 diabetes: Prospective analyses. Health Psychol.

[62] Heffer T, Willoughby T (2017). A count of coping strategies: a longitudinal study investigating an alternative method to understanding coping and adjustment. PLoS One.

